# Improved radiation dose efficiency in solution SAXS using a sheath flow sample environment

**DOI:** 10.1107/S2059798316017174

**Published:** 2016-11-29

**Authors:** Nigel Kirby, Nathan Cowieson, Adrian M. Hawley, Stephen T. Mudie, Duncan J. McGillivray, Michael Kusel, Vesna Samardzic-Boban, Timothy M. Ryan

**Affiliations:** a SAXS/WAXS, Australian Synchrotron, 800 Blackburn Road, Clayton, Victoria 3168, Australia; b B21 SAXS, Diamond Light Source Ltd, Hartwell Science and Innovation Campus, Didcot OX11 0DE, England; c School of Chemical Sciences, University of Auckland, Private Bag 92019, Auckland 1142, New Zealand; d Kusel Design, 6 Hambleton Street, Albert Park, Victoria 3206, Australia

**Keywords:** radiation damage, solution SAXS, sheath flow

## Abstract

Radiation damage is a major limitation to synchrotron small-angle X-ray scattering analysis of biomacromolecules. Flowing the sample during exposure helps to reduce the problem, but its effectiveness in the laminar-flow regime is limited by slow flow velocity at the walls of sample cells. To overcome this limitation, the coflow method was developed, where the sample flows through the centre of its cell surrounded by a flow of matched buffer. The method permits an order-of-magnitude increase of X-ray incident flux before sample damage, improves measurement statistics and maintains low sample concentration limits. The method also efficiently handles sample volumes of a few microlitres, can increase sample throughput, is intrinsically resistant to capillary fouling by sample and is suited to static samples and size-exclusion chromatography applications. The method unlocks further potential of third-generation synchrotron beamlines to facilitate new and challenging applications in solution scattering.

## Abstract 2

Coflow is a new method for delivering radiation-sensitive biological and other solution-based samples to high-brightness X-ray beamlines that exploits laminar flow to ameliorate radiation-damage limitations and provides a host of practical improvements associated with these types of experiments.

## 1. Introduction   

SAXS is a versatile technique for structural biology that is widely applied to confirm high-resolution crystal structures in solution, and provides lower resolution structural and biophysical parameters, particularly for difficult-to-measure protein samples that are not amenable to high-resolution structure determination (for reviews, see Svergun *et al.*, 1995 ▸; Jacques & Trewhella, 2010 ▸). Increasingly, synchrotron SAXS has been advanced through the development of comprehensive data analysis tools, instrumentation and X-ray detectors, and advances in sample presentation and preparation (Graewert & Svergun, 2013 ▸; Bizien *et al.*, 2016 ▸). A major advance in this regard was the development of SEC–SAXS, initially by Mathew *et al.* (2004 ▸), with subsequent incorporation into many synchrotron beamlines (for examples, see David & Pérez, 2009 ▸; Watanabe & Inoko, 2009 ▸; Kirby, Mudie, Hawley, Mertens *et al.*, 2013 ▸). This technique greatly advanced the field, assisting the analysis of mixtures, complexes and polydisperse samples by in-line fractionation, increasing confidence in the monodisperse nature of protein samples entering the beam and simplifying buffer matching. Recent solution SAXS development work has focused on automated sample changers, which have reduced the tedious work of manually obtaining reproducible measurements (for a review, see Round *et al.*, 2015 ▸). These capabilities increase the throughput of the measurements, reduce minimum sample volumes, facilitate high-throughput approaches to research and have expanded the demand for solution SAXS considerably.

X-ray beam damage to biomacromolecules remains a substantial impediment to solution SAXS, particularly on focusing third-generation undulator beamlines where the flux per unit area at the sample position, and thus the dose rates, are very high. Beam damage has largely been mitigated through the use of additives, decreasing flux using attenuators and/or slits, defocusing the beam at the sample position or flowing the sample to distribute dose through a larger volume of sample (Meisburger *et al.*, 2013 ▸; Kuwamoto *et al.*, 2004 ▸; Jeffries *et al.*, 2015 ▸). However, these approaches usually result in a less than optimal data collection efficiency per unit volume of sample. On undulator beamlines, the most common physical methodology to manage beam damage is to flow the sample during analysis. Flow rates are typically well within the laminar flow regime, hence the flow-velocity distribution between the centre and walls of sample cells is highly non-uniform (Reynolds, 1883 ▸). The boundary conditions where the flow velocity approaches zero lead to an extreme radiation dose near the sample cell walls (Gillilan *et al.*, 2013 ▸). Since the sample typically fills the sample cell (*e.g.* capillary, flat thin window cell *etc.*), radiation damage to the sample controls data quality and/or sample consumption under flow analysis. The details of flow and dose distributions are often overlooked, and common practice is to empirically determine levels of reduced incident flux and flow rates that provide adequate results. We present a new approach to solution sample presentation that takes advantage of laminar flow. In the coflow method, the flow of the radiation sensitive sample (*e.g.* protein solution) is constrained to the centre of the sample cell, surrounded by a flow of a matched, radiation resistant solvent (*e.g.* buffer), which is much more able to tolerate extreme radiation doses near the sample cell walls. The new method allows large increases in the incident flux of focused X-ray beams, better matches the sample geometry to the beam size, yields improved data quality and provides several practical advantages that aid routine measurements.

## 2. Modelling the relationship of flow dynamics to radiation dose in capillaries   

**Figure 1:**
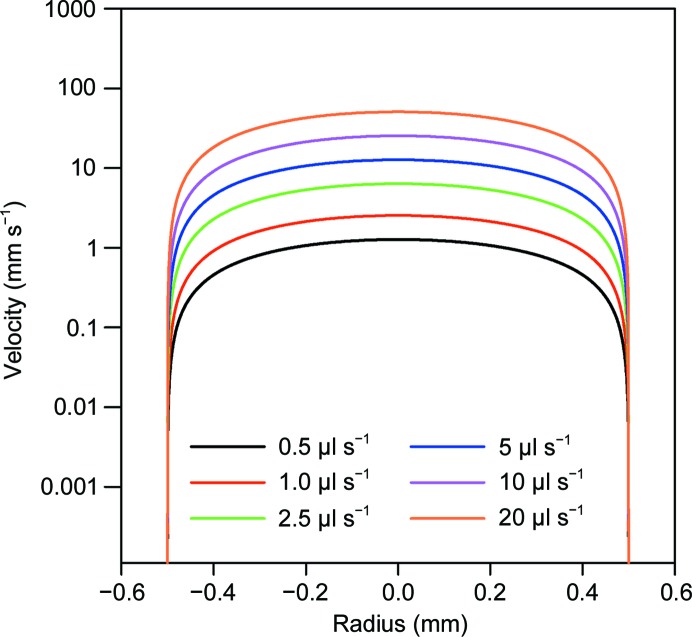
Flow velocity distribution in capillaries. The effective velocity in mm s^−1^ of fluid flow in 1.0 mm capillaries over a range of typical flow rates used for analysis at the Australian Synchrotron (flow rates as indicated).

The flow conditions in the capillary were modelled using the simple parabolic radial velocity profile of laminar flow in a smooth-walled cylindrical tube given by
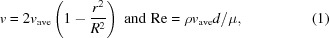
where *v* is the linear flow velocity at a point in the capillary, *v*_ave_ is the average linear flow velocity (in m s^−1^), which is the total volume flow rate (in m^3^ s^−1^) divided by the cross-sectional area (in m^2^), *r* is the radial distance from the centre of the capillary, *R* is the internal radius of the capillary wall, Re denotes the Reynolds number, ρ is the density of the fluid (in kg m^−3^), *d* is the internal diameter of the capillary (in m) and μ is the dynamic viscosity of the fluid (0.89 × 10^−3^ Pa s for water at 25°C). The Reynolds number for a 1 µl s^−1^ flow of water in a 1 mm diameter capillary is 2.4, with an average velocity 2000 times lower than the critical velocity for breakdown of laminar flow (Reynolds, 1883 ▸). Hence the flow regime is laminar, even for flow rates that are one to two orders of magnitude higher than those typically used for static protein and SEC analyses. The flow field in the capillary is highly non-uniform, as under a laminar flow regime there is a parabolic flow velocity distribution, stretching from zero at the interface between the capillary and the solution to a maximum at the centre of the capillary of twice the average velocity (Fig. 1 ▸). The X-ray dose experienced by a protein molecule is proportional to its dwell time in the beam; hence, the flow-velocity distribution has a strong effect on the dose distribution.

A quantitative numerical model based on this flow field was developed to determine the X-ray dose distribution in horizontal circular capillaries in Gaussian X-ray beams under laminar flow conditions. This two-dimensional pixel-by-pixel model takes into account the beam intensity profile and flow velocity distributions, and the sample absorption, rather than simply averaging over the FWHM (full width at half maximum) or full beam width. One half of a circular capillary of any known diameter (the analysis was assumed to be laterally symmetric) was divided into a 500 (H) × 1000 (D) grid of square pixels. The incident, absorbed and transmitted power through each pixel was calculated from the Gaussian horizontal beam-intensity profile measured using a calibrated photodiode and corrected for capillary absorption. The absorption in the fluid was calculated using the Beer–Lambert law and the attenuation coefficient of water to approximate those of common aqueous buffers. The absorbed dose in grays for each pixel was calculated from the absorbed power, the cross-sectional area and the calculated flow velocity at the centre of each pixel, and the density and the linear attenuation coefficient of the solvent. The model calculates the dose distributions of the whole capillary, and for sheath flow the radius and dose distribution of the sample stream. Radiation damage observed in a SAXS measurement is subject to the net contribution of each pixel to the scattering pattern, which is proportional to the flux transmitted through each pixel that reaches the X-ray detector. The scattering intensity weighted average dose was calculated by linearly weighting the dose absorbed by each pixel according to the flux transmitted through each corresponding longitudinal column of pixels.

The boundary condition of zero flow velocity at the stationary interface results in doses that approach infinity. Minute changes in model parameters stochastically affect the centroid location of pixels in a rectangular array modelling a circular or arbitrary sample-cell geometry. Small deviations in the centroids of extreme boundary pixels produce excessive variations in calculated velocities and thus dose. Despite their almost insignificant volumetric contribution to the overall scattering pattern, the calculated doses in small numbers of boundary pixels can be so high that they can artificially bias the calculated average dose results by as much as 10–30%. We overcame this spatial resolution limitation by excluding all pixels directly contacting the wall of the capillary from the model. For most pixels, the dwell time in the beam (*e.g.* the vertical FWHM divided by the linear velocity) is considerably shorter than the open time of the shutter in many practical measurements, and the dose can be calculated from the absorbed power and flow rates assuming infinite exposure times. However, for very slowly moving regions nearer to the edges of the capillary, the dwell time in the beam calculated from the flow velocities may be longer than the time that the shutter is actually open. Thus, a finite total exposure time was accounted for in the dose calculations of all pixels by limiting the minimum flow velocity of any pixel to the vertical FWHM of the beam divided by the total exposure time. The final numerical model can calculate sample diameter, velocity and dose distribution, average dose, sample and solvent scattering intensities and scattering intensities per volume of sample. The boundary conditions in a finite-resolution model imply some approximation of buffer doses in very close proximity to the capillary surface, but have no significant effect on calculated doses of the protein stream, which are the focus of this work.

**Figure 2:**
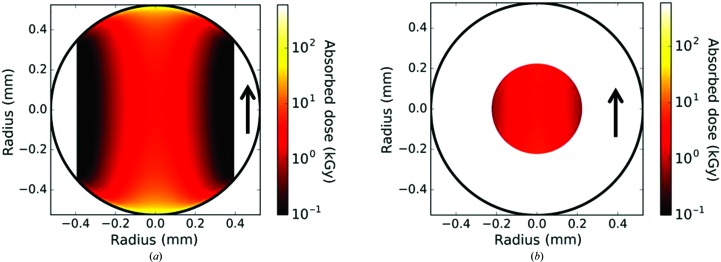
Radiation dose modelling. (*a*) Pixel-based numerical modelling of the sample-absorbed dose under conventional flow conditions at a flux of 5 × 10^12^ photons s^−1^. Note that the absorbed dose in the longitudinal (*i.e.* along the beam) direction is dominated by flow-field effects, while the absorbed dose in the lateral (*i.e.* perpendicular to the beam) direction is dominated by the Gaussian beam profile. The absorbed dose varies by several orders of magnitude from the front to the middle of the capillary, reflecting the increased time in the beam for material in the low-flow regions. (*b*) Calculated sample-absorbed radiation dose in the coflow measurement, using a total flow of 1.0 µl s^−1^ and a fractional sample-flow rate (FSFR) of 0.33 at 5 × 10^12^ photons s^−1^, indicating a much more uniform and lower absorbed dose restricted to the central portion of the capillary. The arrows indicate the beam direction.

Applying the model to the standard conventional capillary measurement indicated that the dose distribution in a flowing capillary illuminated by a small Gaussian X-ray beam is highly non-uniform (Fig. 2 ▸). The longitudinal dose profile is dominated by the flow velocity profile and ranges over several orders of magnitude, while the lateral dose distribution is dominated by the lateral X-ray beam profile. Absorption for 1 mm capillaries with aqueous buffers at 12 keV also plays a role in the shape of the dose distribution, albeit to a lesser extent than the beam profile and fluid dynamics. In the centre of the beam, the front and back regions near the capillary wall receive radiation doses that are far in excess of the average dose across the capillary. For conventional flow analysis where the capillary is full of protein solution, these regions are likely to dominate the radiation damage of proteins and fouling of the capillary by radiation damaged sample. Reducing damage in these regions by increasing the flow rate or reducing the flux will result in significant average under-dosing of samples. It is also worth noting that the portion of the sample flowing outside the lateral FWHM of the beam is measured ineffectively or not at all.

Unsurprisingly, the dose distribution at the centre of the capillary was relatively flat, supporting the idea of introducing a sample stream inside a sheath flow of buffer. Modelling of various flow rates, fractional sample flow rates, incident flux, capillary diameters and beam sizes showed obvious improvements in sample dose per incident flux compared with conventional flow analysis at equal sample flow rates, and suggested overall improvements in data quality per sample volume despite the reduced sample path length. Under laminar flow, it was anticipated that stable, nonmixing flow over adequate distances and timescales could be readily achieved. Given the results from modelling, a dedicated sample cell was designed and constructed to test a sheath flow regime for SAXS measurements.

## 3. The sample cell and sample measurements   

### 3.1. The sample cell   

**Figure 3:**
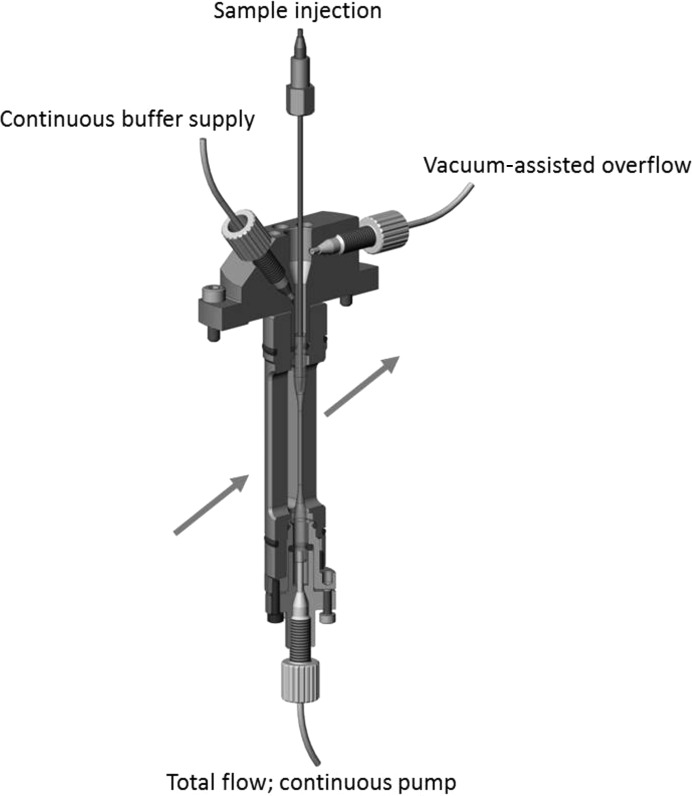
CAD drawing of the coflow cell. The stainless steel assembly holds a custom 1 mm double-ended capillary which is sealed through O-ring compression by the top and bottom mounts. The outer O-rings provide a seal for in-vacuum (windowless) analysis if required. The top mount is open, allowing a needle to be positioned for submarine loading, and has connections for a continuous buffer supply, if needed, and a vacuum-assisted overflow port. The bottom provides the port for the total flow pump, which controls the total flow of buffer and sample through the capillary. At this stage of development the total flow was controlled by a VICI M50 displacement pump, and sample flow was driven by a Hamilton PSD-8 syringe pump. The arrow indicates the beam direction.

To generate a sheath flow, we designed a custom sample cell. We have termed the technique of generating a sheath flow in SAXS analysis ‘coflow’. The coflow sample cell design and plumbing connections are shown in Fig. 3 ▸. The cell is designed for use in vacuum but, at this stage of development, all data presented here were collected using the cell in air. The sample cell consists of a stainless steel outer assembly, into which a custom hourglass-shaped quartz capillary (Hilgenberg) with thick wall openings at both ends is sealed with lightly compressed O-rings. The central portion of the capillary is 1 mm in diameter with 50 µm thick walls. The bottom of the capillary is connected to a continuous-flow pump (*e.g.* VICI M50 series) which controls the total flow rate out of the sample cell. The capillary is filled with buffer, and for most continuous operations (*i.e.* SEC or large groups of samples with the same buffer) the level of buffer is maintained by a second continuous-flow pump attached to an inflow port at the top of the housing. Overflow is removed by a vacuum take-off port also located at the top of the housing. The top of the cell is open, allowing easy access for the HPLC autoloader-based sample needle (Agilent). This needle is located in the centre of the capillary and is positioned 1–2 mm above the beam position, well under the meniscus of the buffer. This arrangement removes any requirement for a mechanical seal for sample loading, and aids in both rapid loading and the analysis of very small sample volumes. Additionally, the open top is useful for washing and discontinuous buffer flow, and allows rapid and efficient buffer exchange. The volume of buffer at the top of the cell is more than sufficient for a single sample or a short concentration series. As the flow progresses, the buffer is drawn down the outside of the needle and surrounds the sample outflow from the sample needle in the centre of the capillary, generating a concentric sheath flow. In this case, the outer buffer flow rate is not controlled directly by a pump but is the difference between the total outflow and sample inflow rates.

**Figure 4:**
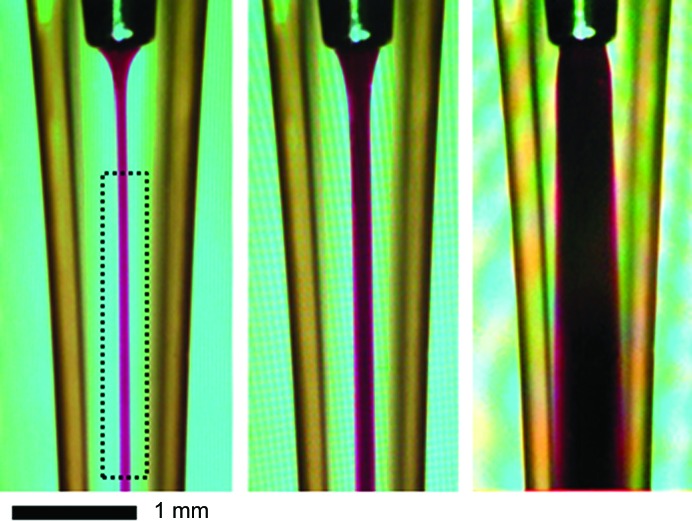
Visual demonstration of stable laminar flow in the coflow cell using a dye solution as a sample and water as a sheath flow, and the effect of increasing the FSFR from 0.05 (left panel) to 0.1 (central panel) and 0.8 (right panel). The dotted box indicates a suggested beam position; however, the beam can be located anywhere from ∼1 mm from the end of the needle to the bottom of the capillary (∼2 cm), with no apparent changes in the data acquired, apart from minor variation in the thickness of the capillary. The taper apparent in the image is owing to drawing errors in this particular capillary. The scale bar indicates a distance of 1 mm in this image.

This apparatus creates a stable laminar flow that persists for centimetres along the capillary at flow rates appropriate to an undulator beamline (Fig. 4 ▸). The velocity profile across the capillary is determined by the total flow rate out of the capillary. The sample diameter is controlled by the rate of the sample flowing in from the injection needle as a fraction of the total flow rate out of the capillary (fractional sample flow rate; FSFR). Using sample radii well above diffusion lengths for the time interval between the injection and analysis position prevents significant mixing of sample and buffer. By using matched buffer in the outer flow stream, the accuracy of background subtraction of the conventional method is preserved at all times, even for non-steady-state sample flow.

It is of critical importance to the stability of the SAXS measurement that the sample injection and outflow pumps deliver accurate, smooth, non-pulsatile flow. We used a Hamilton PSD-8 fitted with a 50 µl syringe driven by an external motor controller microstepping at 25 000 steps per motor revolution for sample delivery. Continuous-flow pumps are more convenient for the outflow and buffer flow lines: VICI M50 displacement pumps with the same microstepping were used for this development work.

### 3.2. SAXS measurements in the sample cell   

SAXS data were acquired on the SAXS/WAXS beamline at the Australian Synchrotron (Kirby, Mudie, Hawley, Cookson *et al.*, 2013 ▸), which has been optimized for low instrument background. Data were acquired using camera lengths of 2.680 or 1.426 m, providing *q*-ranges of 0.004–0.25 or 0.01–0.5 Å^−1^, respectively. All data presented were acquired at 12 keV using continuous exposures read out on the fly, with individual integration times from 0.25 to 1 s (as noted) and an FWHM beam size of 130 × 250 µm (vertical × horizontal). Samples can be static aliquots (*e.g.* loaded from well plates) or a continuous flow from size-exclusion chromatography or mixing devices. Similar to the conventional analysis, static samples should be well matched to buffer, *e.g.* by dialysis. For static samples, a volume of buffer is injected from the autoloader needle into the top of the capillary. The output flow pump is then started and once buffer is flowing through the X-ray beam, continuous acquisition of scattering data commences. After acquiring sufficient buffer data, the sample is injected at a controlled flow rate into the centre of the capillary just above the X-ray beam position. Data acquisition continues at least until the sample has finished traversing the beam. Data collected during steady-state sample flow are used for routine analysis because they have the highest and most consistent scattering intensity, and moreover can be processed using a fixed absolute scaling calibration for a given fractional sample flow rate. Data collected before and after steady-state sample flow can be screened out in software. If another sample is to be analyzed in the same buffer it can be loaded as soon as a loading needle is in position to inject it, without cleaning the capillary in between sample additions. If the buffer is to be changed, the remaining solution can be rapidly drawn out of the capillary by the total flow pump, the reservoir flushed with water if desired and fresh new buffer loaded in a straightforward and simple operation. Given the small minimum sample volume requirement of coflow, it is essential for static samples to wash and thoroughly dry the loading needle and loop after sample measurement to prevent crossover or dilution of samples or buffers. Bubbles, if introduced or formed, can disrupt the laminar flow of the system and must be avoided. Particular care should be given to exhaustively degassing samples and buffers prior to use, as the high flux of the beam can cause absorbed gases to be released from solutions. Should bubbles be detected, they can be removed in various ways, such as by a fast flow of buffer, draining the capillary, stopping flow and withdrawing the loading needle, and/or washing/drying the system.

For SEC analysis, buffer is pumped continuously into the buffer port at the top of the cell, the sample is injected continuously through the needle and both are drawn through the capillary using the output pump. Buffer supplied in slight excess avoids having to exactly match the total inflow and outflow rates. Buffer is exchanged by changing input supplies and flushing the system.

Finally, the setup supports conventional sample presentation without modification by only using sample flow.

### 3.3. Absolute calibration and data analysis   

**Figure 5:**
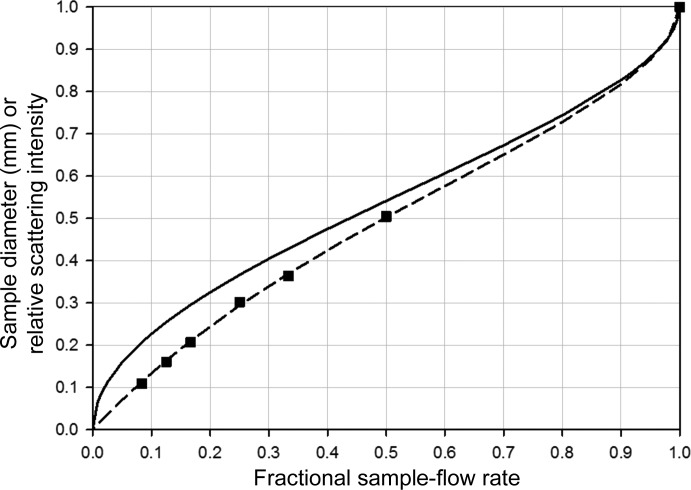
Comparison of the theoretical dilution effect to experimental effective dilution. Effect of a fractional sample flow rate in a 1 mm diameter capillary on the diameter of the inner sample flow (solid line), and the calculated (dotted line) and experimentally observed (squares) scattering intensity of the sample relative to conventional flow analysis.

The diameter of the central sample stream depends on the fractional sample flow rate (FSFR) of the total flow rate. Since the sample occupies only part of the total fluid X-ray path length, but the solvent fills the full path length, the ratio of sample to solvent scattering is reduced. This is similar to dilution as there is reduced sample scattering without changing the solvent scattering intensity. Thus. we have denoted this effect as ‘effective dilution’ even though no significant mixing between the sample and buffer flows occurs (Fig. 4 ▸). The effective dilution effect must be taken into account for absolute intensity calibration. Water is convenient for calibrating the absolute scattering intensity calibration of the full cell path length. In practice, the best results appear to come from fixing the FSFR once optimized for the application: our setup has given good results for FSFRs between 0.3 and 0.5. The settings could be varied to suit different capillary diameters, beam profiles and individual preferences. For a well aligned cell in the centre of the beam with a well known intensity profile the effective dilution under steady-state flow agrees to within 5% of that calculated from the flow geometry and beam profile (Fig. 5 ▸); hence, a full-cell water empirical calibration and calculated correction factor is adequate for most measurements. Alternatively, a fully empirical calibration can be performed using a protein standard of known concentration at the FSFRs of interest. For samples that are too small to produce steady-state flow (*e.g.* <2 µl), absolute intensity calibration requires an empirical protein standard.

All SAXS images were inspected, averaged and subtracted using *scatterBrain* v.2.82 (http://www.synchrotron.org.au/aussyncbeamlines/saxswaxs/software-saxswaxs). Reduced data in the form of data files were subjected to pairwise comparison using *DATCMP* (Petoukhov *et al.*, 2012 ▸), with a Student t-test, Bonferroni post-hoc correction, *p*-value level of 0.05, to determine the presence of radiation damaged material. Basic data analysis using Guinier plots to calculate the radius of gyration was conducted using *AUTORG* from the *ATSAS* suite of SAXS data-analysis tools (v.2.7.1; Petoukhov *et al.*, 2012 ▸). Further Guinier analyses, and statistical assessments of beam damage, were conducted using the nonlinear regression and t-test functionality of *SigmaPlot* 13. Uncertainties from Guinier fits are two standard errors of the slope of fitted linear regressions of ln(*I*) *versus* *q* ^2^. Data quality and accuracy were assessed using *CRYSOL* (Svergun *et al.*, 1995 ▸) to compare the acquired data with calculated scattering curves for known crystal structures for RNase A and glucose isomerase. Calculation of the Porod volume was conducted using *DATPOROD*, and *I*(0) and *D*_max_ values were obtained from *DATGNOM*, both of which are included in *ATSAS* v.2.71 (Petoukhov *et al.*, 2012 ▸). Molecular weight was calculated according to the method of Fischer *et al.* (2010 ▸) and was confirmed using the method of Rambo & Tainer (2013 ▸).

## 4. Performance of the coflow cell and considerations for high-flux solution measurements   

### 4.1. The effect of high dose on buffer components   

**Figure 6:**
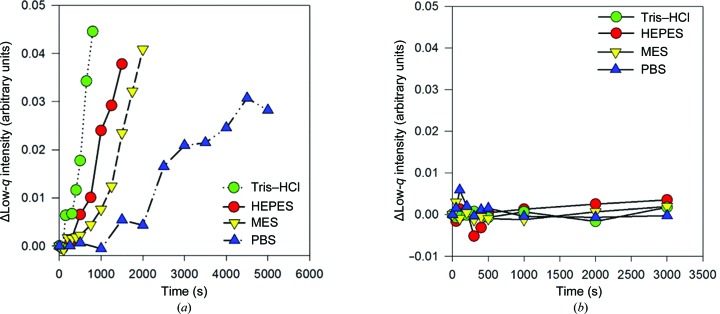
Radiation damage to buffer components. (*a*) The change in low-*q* intensity (average *q* between 0.005 and 0.008 Å^−1^) as a function of time, showing the relative radiation resistance of buffers (as indicated in the figure legend; the actual data are given in Supplementary Fig. S1). (*b*) Addition of glycerol prevents radiation damage to buffers. The change in low-*q* intensity (average *q* between 0.005 and 0.008 Å^−1^) highlights the increased radiation resistance of buffers upon the addition of glycerol (buffers as indicated in the legend; data are given in Supplementary Fig. S2).

Our modelling indicates that coflow allows a considerably higher incident flux before resulting in the equivalent average dose to the protein solution as conventional flow analysis. This is one of the main benefits, but it also is a potential drawback because the buffer, which is still subject to slow flow at the edges of the capillary, must withstand very high doses. There is little comprehensive work on the tolerance of common buffer components to high-flux X-rays. Thus, as a first step towards characterizing the behaviour of samples in coflow, the effect of extended continuous exposure (4 × 10^12^ photons s^−1^ at 12 keV, 220 × 130 µm FWHM H × V beam) on four common pH 7.5 buffers at total flow rates of 1 µl s^−1^ in the 1 mm diameter capillary was investigated (Fig. 6 ▸ *a*, Supplementary Fig. S1). All showed time-dependent changes within tens of minutes of continuous exposure. PBS displayed the most resistance, followed by MES and then HEPES; Tris–HCl was the least resistant. The radiation damage products adhered to the capillary wall, were not removable by washing and gave SAXS patterns consistent with aggregated or polymerized material. This degree of damage and accumulation was certainly sufficient to impede routine SAXS measurements.

Radiation damage to proteins in solution may be reduced by the addition of 2–5% glycerol (Jeffries *et al.*, 2015 ▸; Kuwamoto *et al.*, 2004 ▸), an additive that is suitable for a wide range of biological samples. Hence, we investigated whether 5% glycerol would improve the radiation resistance of the buffers themselves (Fig. 6 ▸ *b*, Supplementary Fig. S2). Glycerol improved the dose tolerance of the four buffers at least 200-fold, allowing continuous measurement over hours of full flux on this beamline. Although buffer damage at lower flux levels may be subtle or go unrecognized in conventional synchrotron measurements, it is critically important to avoid it at the high-flux levels used for coflow, particularly for HEPES and Tris–HCl. In all subsequent measurements, the buffer contained 5% glycerol unless otherwise stated.

### 4.2. Improvement of dose efficiency   

The capacity of coflow to use an increased X-ray flux was quantified from measurements on ribonuclease A (RNAse A). Previous research estimated that the critical dose for damage of RNAse A in PBS is well within the dose range accessible to both coflow and conventional analysis on this beamline (Jeffries *et al.*, 2015 ▸). In the presence of glycerol, the radiation resistance of RNAse A is increased (∼4 kGy; Jeffries *et al.*, 2015 ▸) enough to make it difficult to cause substantial radiation damage in coflow geometry on this beamline despite using the full X-ray flux. Thus, RNAse A was prepared by suspending 10 mg of powder in 2 ml of glycerol-free PBS, which was then dialysed against 1 l of this buffer for at least 24 h prior to the measurement. The dialysis buffer was used for buffer-subtraction measurements. The protein concentrations were determined using UV absorbance and an extinction coefficient at 280 nm of 13 870 *M* ^−1^ cm^−1^, giving a concentration of 4.92 mg ml^−1^. Coflow analysis was performed in the absence of glycerol using an FSFR of 0.33 and a total flow rate of 1 µl s^−1^, while conventional analysis used a 1 µl s^−1^ sample-flow rate. A flux ranging from 7 × 10^10^ to 5 × 10^12^ photons s^−1^ was used to vary the X-ray dose.

**Figure 7:**
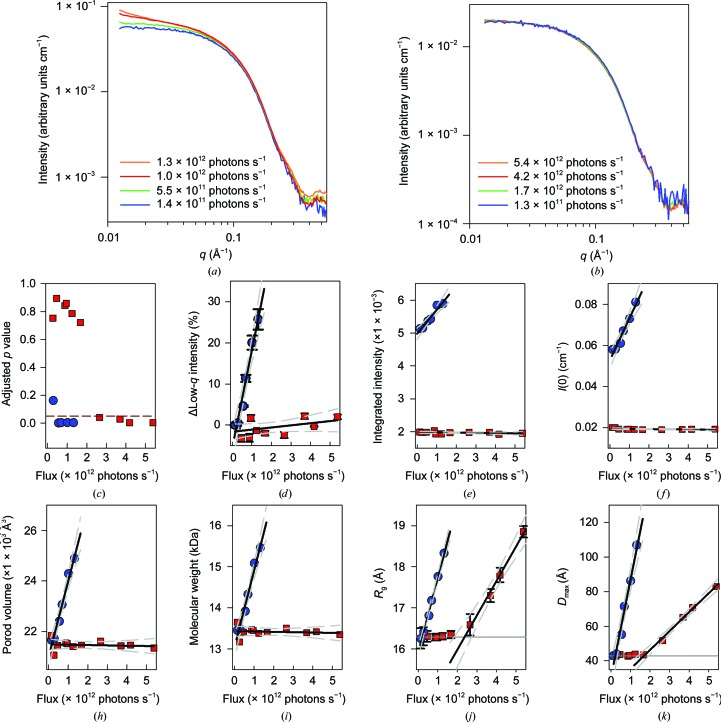
Comparison of radiation damage in coflow *versus* conventional analysis. The effect of X-ray flux on RNase A scattering in conventional (*a*) and coflow (*b*) sample environments. In the conventional measurement RNase A formed aggregates, as displayed by the large increase in scattered intensity at *q* < 0.025 Å^−1^ in (*a*). In coflow, aggregation did not occur. A variety of parameters, including the statistical similarity of curves (calculated with *DATCMP*; the red dashed line indicates a *p* value of 0.05) (*c*), average low-*q* intensity (*d*), integrated intensity (calculated as described by Hopkins & Thorne, 2016 ▸) (*e*), *I*(0) (obtained from *DATGNOM*) (*f*), Porod volume (obtained from *DATPOROD*) (*g*), molecular weight (calculated as described by Fischer *et al.*, 2010 ▸) (*h*), radius of gyration [from linear regression of Guinier plots in *SigmaPlot* 13 (shown in Supplementary Fig. S3); the solid grey line indicates our observed *R*_g_ for undamaged RNase A of 16.2 ± 0.2 Å] (*i*), maximum particle size (*D*_max_, from *DATGNOM*; the solid grey line indicates the undamaged RNase A *D*_max_ of 42.8 ± 0.5 Å) (*j*), indicate large differences between conventional (blue circles) and coflow (red squares) in the response of RNase A to increasing flux. The solid black lines indicate linear regression of the increases in the various parameters; the dashed grey lines indicate 95% confidence intervals of the linear fit.

In the conventional method, RNAse A showed increasing aggregation with increasing flux (Fig. 7 ▸ *a*). Pairwise comparison of the SAXS patterns using *DATCMP* indicted that the data acquired with a flux greater than 5.5 × 10^11^ photons s^−1^ were significantly different from the initial low flux data (Fig. 7 ▸ *c*). Further analysis of the average low-*q* intensity, *I*(0), Porod volume, molecular weight, *R*_g_ (through Guinier plots; Supplementary Fig. S3) and *D*_max_ (Figs. 7 ▸ *d*–7 ▸ *i*) all varied positively with increasing flux. The critical flux was estimated from linear regression of the dependence of *R*_g_ on flux in the region where the *R*_g_ increased. The critical flux and its uncertainty were taken as the flux at which the linear regression and its 95% confidence intervals, respectively, intersected the undamaged data (16.2 Å). The critical flux observed under these conditions for conventional analysis was 2.1 × 10^11^ ± 5 × 10^10^ photons s^−1^.

In contrast, the data collected using coflow showed no sign of aggregation at even the highest flux (Fig. 7 ▸ *b*). However, analysis with *DATCMP* indicates that the data collected using a flux greater than ∼2.5 × 10^12^ photons s^−1^ were significantly different from the initial low flux data set. Analysis of the average low-*q* intensity, *I*(0), Porod volume and molecular weight indicated that these parameters were independent of increasing flux when measured using coflow (Figs. 7 ▸ *d*–7 ▸ *h*). The only parameters to show significant change with increased flux were the *R*_g_ and *D*_max_. The analysis of the *R*_g_ data indicated a critical flux of 2.4 × 10^12^ ± 4 × 10^11^ photons s^−1^ (Figs. 7 ▸ *i* and 7 ▸ *j*), above which results indicate conformational change but not aggregation. Compared with the critical flux for the conventional method, coflow showed an 11-fold increase in useable incident flux at the same total flow rate. When the difference in sample-flow rates between conventional (1 µl s^−1^) and coflow (0.33 µl s^−1^) is taken into account there is a 31-fold increase in applied flux per sample volume. This efficient dose delivery can be used to reduce the sample volume or increase the data quality, both of which are particularly useful to biological researchers.

### 4.3. The coflow geometry reduces sample-volume requirements   

**Figure 8:**
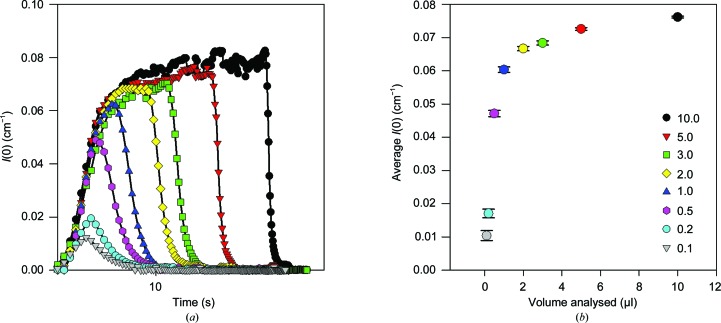
The dependence of *I*(0) on the volume of analyte. (*a*) Samples of GI (1.5 mg ml^−1^) ranging in volume from 0.1 to 10 µl were analysed by coflow using a total flow of 1 µl s^−1^ and an FSFR of 0.33, with data collected every 0.5 s. *I*(0) was calculated from the linear regression of the Guinier plot. (*b*) The average *I*(0) was calculated by calculating the mean of the plateau region for the data sets in (*a*) [or peak *I*(0) for ≤1 µl] and plotting as a function of the volume of analyte. Error bars indicate two standard errors of the average *I*(0).

By directly injecting the sample from a moving loading needle rather than pumping it through tubing, coflow allows the possibility of analyzing very small static sample volumes. A 1.5 mg ml^−1^ glucose isomerase (GI) solution, prepared by dialyzing 1 ml of a 33 mg ml^−1^ stock in 6 m*M* Tris–HCl, 1 m*M* MgSO_4_, 120 g l^−1^ ammonium sulfate pH 7.0 (Hampton Research) against 1 l of 10 m*M* HEPES, 1 m*M* MgCl_2_, 5% glycerol pH 7.5 for 24 h and then diluting to the appropriate concentration, was used as a test sample. The dialysate was used as a diluent and as the buffer blank for the SAXS measurements. Protein concentrations were determined using the absorbance at 280 nm and an extinction coefficient of 45 666 *M* ^−1^ cm^−1^. The minimum sample volume was determined by injecting 0.1–10 µl aliquots of 1.5 mg ml^−1^ GI and monitoring the change in *I*(0) over time (Fig. 8 ▸ *a*). Sample scattering intensity rose rapidly after injection started. If a sufficient sample volume was injected, the scattering intensity then reached a steady state. Once injection ceased, the sample scattering decreased to zero as the trailing end of the sample passed the beam position. Quantitative scattering intensities are most easily measured during steady-state flow, which required a minimum sample volume of 2 µl under these conditions. This gave an average *I*(0) of ∼0.070 ± 0.02 cm^−1^ and a concentration-normalized *I*(0) of 0.046 cm^−1^ mg^−1^ ml. Smaller sample volumes did not achieve steady-state flow, and their peak *I*(0) decreased in a roughly linear fashion with sample volume (Fig. 8 ▸ *b*). Although sample path lengths and thus absolute scaling below 2 µl may be qualitative, background subtraction remained accurate because coflow with a matched buffer maintains a fixed solvent path length. All data sets, including the 0.1 µl data, had an *R*_g_ of 32.8 ± 0.5 Å, consistent with that of GI obtained by conventional analysis (Kirby, Mudie, Hawley, Cookson *et al.*, 2013 ▸).

These data indicate that coflow supports a minimum sample volume of 2 µl with quantitative absolute scaling. This volume is 25-fold lower than the routine minimum sample required by the conventional autoloader and sample presentation method­ology as implemented at the Australian Synchrotron (Kirby, Mudie, Hawley, Mertens *et al.*, 2013 ▸). With the current equipment and capillary geometry, coflow can accommodate sample volumes below 2 µl, producing accurately background subtracted data without quantitative absolute scaling, with measurement statistics adequate for perhaps *R*_g_ determination only.

### 4.4. Coflow achieves comparable detection limits and improved data quality   

**Figure 9:**
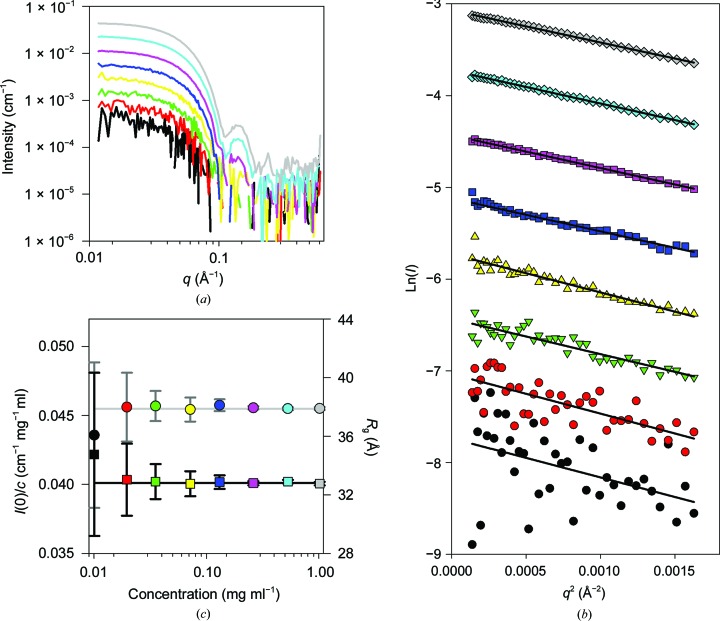
Sensitivity/limits of detection of the coflow method. (*a*) Extended serial dilution series of glucose isomerase at 1.0 (grey), 0.50 (light blue), 0.25 (pink), 0.125 (dark blue), 0.0625 (yellow), 0.0313 (green), 0.0152 (red) and 0.0075 mg ml^−1^ (black). Data are binned in logarithmic *q* space. (*b*) Guinier fits of the data in (*a*) were conducted by linear regression of the data to the Guinier equation using *SigmaPlot* 13, which provides confidence intervals and standard errors of the slope of the fit, and hence the standard error of the radius of gyration and *I*(0). Data sets are offset by 0.5 units for clarity. (*c*) Observed radius of gyration, *R*_g_ (squares), and concentration-normalized intensity at zero *q*, *I*(0) (circles), are plotted *versus* concentration for serial dilution series of GI. The light grey line indicates the *I*(0) for 1 mg ml^−1^ GI (0.0455 cm^−1^). The dark grey line indicates the *R*_g_ for 1 mg ml^−1^ GI (32.8 Å). Uncertainties are ±2 standard errors of the slope of linear regression.

We investigated coflow detection limits and sensitivity on a GI dilution series (prepared as described) between 0.008 and 1.0 mg ml^−1^. The data and subsequent results of Guinier analysis are shown in Fig. 9 ▸, indicating that accurate *R*_g_ values (the expected *R*_g_ is 32.5 Å) were obtained at all measured concentrations. This is comparable to conventional analysis despite using only one tenth of the sample volume used in the previous study (Kirby, Mudie, Hawley, Cookson *et al.*, 2013 ▸). The *I*(0) in coflow is lower than that of the conventional measurement because of the reduced sample path length. This reduction of ∼2.5-fold is comparable to the ‘effective dilution’ calculated for an FSFR of 0.33 based on the flow, capillary and beam geometry.

The *I*(0) normalized to concentration indicates a remarkably consistent value of 0.045–0.047 cm^−1^ mg^−1^ ml. *CRYSOL* fitting of the concentration series to the GI tetramer crystal structure (PDB entry 1oad) demonstrates that all data sets accurately represented the expected GI tetrameric structure within measurement statistics (Supplementary Fig. S4) to concentrations comparable to previous conventional measurements on this beamline. This indicates that the coflow method is routinely applicable to samples with concentrations down to ∼0.05 mg ml^−1^ depending on the molecular weight and the information required. Owing to the geometry of the coflow, and the fact that a constant stream of buffer is running through the system regardless of sample flow, buffer data can be collected frequently. Further, the geometry forces the collection of buffer data in close time proximity to the sample data collection. Both of these features aid in the accuracy of buffer subtractions, as the close time proximity reduces the chance of instrument instability affecting the buffer data and the frequent collection can be used to reduce the amount of noise present in the low-signal buffer scattering data.

Detailed data analysis using four methods of comparing the statistical data quality in the Guinier region shows that coflow halved random errors for the same sample volume and buffer analysis time of RNase A data at the critical flux determined in §[Sec sec4.3]4.3. The average percent Poisson counting error [100 × *I*(*q*)/*N*(*q*)^1/2^], where *I* is the background-subtracted scattering intensity and *N* is the number of counts in each *q* bin recorded on the photon-counting detector, was 0.49% for coflow and 0.99% for conventional analysis across the Guinier region. The Poisson statistical uncertainty of coflow relative to conventional analysis, estimated from the 9.6-fold increase in total photons used for background measurement and the 27-fold increase in total photons used for sample measurement, but taking into account the 2.6-fold effective dilution owing to sample-pathlength reduction, was 46%. The 95% confidence interval of the Guinier slope for coflow was 46% that of conventional analysis. The coefficient of variation of the residuals of the observed intensities from the Guinier fit was 0.57% compared with 1.1%, showing that observed improvement in random errors of coflow match those calculated from measurement statistics. Thus, coflow significantly improves data quality, while maintaining a sensitivity and accuracy comparable to conventional flowing SAXS analysis.

### 4.5. The sheath flow abrogates capillary fouling   

**Figure 10:**
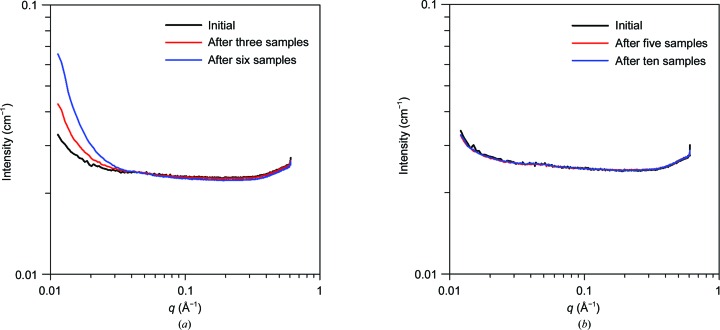
Abrogation of capillary fouling in coflow. Buffer data sets (1 s shots averaged over 30 s total exposure) before any sample is measured (black line), in the middle (red line) and after (blue line) the RNase A damage series in Fig. 8 ▸ for conventional (*a*) and coflow (*b*) sample environments.

In the conventional flow approach the sample is in direct contact with the capillary, and thus unstable samples may form deposits anywhere along the capillary. Of particular concern is that the highest dose occurs in close proximity to the capillary, which exacerbates fouling by radiation damage products. This results in the all-too-often observed buildup of damaged proteins at the beam position of focused undulator beamlines. While software-based approaches to deal with this have been developed in regards to SEC analysis (Brookes *et al.*, 2016 ▸), there is no physical method for reducing this accumulation. Coflow prevents contact between the sample and the capillary near the point of X-ray analysis, removing this as an obstacle to routine operations. In practice, as long as the sheath flow is maintained, fouling by the sample is eliminated (Fig. 10 ▸), even when deliberately overdosed to cause severe radiation damage to the protein (damage shown in Figs. 7 ▸ *a* and 7 ▸ *b*).

## 5. Discussion and conclusions   

Radiation damage is a major limiting factor in the advancement of SAXS analysis of solution samples. Previous studies on radiation damage have focused on a static sample, which removes complexities arising from very wide dose distributions owing to flow profiles (Hopkins & Thorne, 2016 ▸; Jeffries *et al.*, 2015 ▸; Kuwamoto *et al.*, 2004 ▸; Meisburger *et al.*, 2013 ▸). These studies provide important frameworks with which to understand critical dose levels, assess damage and avoid damage (Jeffries *et al.*, 2015 ▸; Hopkins & Thorne, 2016 ▸; Kuwamoto *et al.*, 2004 ▸), including the use of stabilizing additives. A novel cryogenic method to abrogate radiation damage and greatly increase data quality per sample volume has also been reported. However, this approach requires large additions of PEG to prevent ice crystallization on rapid cooling and has been reported to have difficulties with buffer subtraction at high *q* (Meisburger *et al.*, 2013 ▸). Unfortunately, the study of static conditions provides limited insight into how flow dynamics affect the absorbed radiation dose. Fluid dynamics, and the influence of Navier–Stokes-based modelling of fluid flows in SAXS capillaries, have been investigated by Gillilan and coworkers (Gillilan *et al.*, 2013 ▸; Nielsen *et al.*, 2012 ▸). They postulate that the nonslip boundary indicated by this modelling will result in a layer of radiation damaged material that is impossible to remove under steady-flow conditions (Gillilan *et al.*, 2013 ▸). The solution proposed was an oscillating flow cell, which redistributes radiation-damaged material, allowing the replenishment of undamaged sample (Nielsen *et al.*, 2012 ▸). The results of the pixel-based modelling and experimental measurements of the effect of sheath flow on radiation damage, dose and data quality is an extension of these studies. The pixel-based modelling provides a practical tool for determining the absorbed dose distribution in a flowing solution under cylindrical laminar-flow conditions. This may be of use for designing improved geometries for solution based SAXS measurements.

The coflow buffer sheath approach is a novel solution for improving radiation dose efficiency that is applicable across standard temperature and *in vitro* solution chemistry conditions. This offers numerous improvements in measurement quality and practical considerations. Calculations and empirical results show that increased sample dose uniformity allows an at least tenfold higher incident flux, which outweighs the modest reduction in sample path length inside the cell. The method maintains adequate sensitivity for measuring dilute samples and improves data quality (signal-to-noise ratio) per sample volume relative to the conventional flowing capillary method.

**Figure 11:**
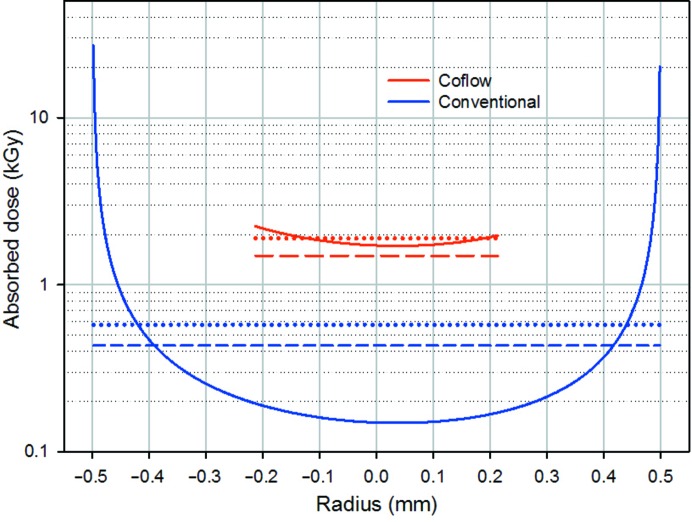
The effect of non-uniform dose on critical flux. Cross-section through the absorbed dose distribution at critical flux experimentally observed from *R*_g_ for RNAse A for conventional (2.1 × 10^11^ photons s^−1^) and coflow analysis excluding the buffer region (2.4 × 10^12^ photons s^−1^) at 12 keV using a 1 mm diameter capillary; FSFR = 0.33. Solid lines are the local dose absorbed by pixels in the horizontal centre of the beam, plotted as a function of their distance from the centre of the capillary (*i.e.* the front and back of the capillary at −0.5 mm and 0.5 mm, respectively). The dotted and dashed lines are the scattering intensity weighted average dose in the centre of the beam and over the full beam-interaction volume, respectively.

These empirical results provide potential insights into the nature and consequences of radiation damage. Firstly, we observe that because of non-uniform dose distributions, static, coflow and conventional flow analysis do not directly assess the fundamental dose limit of the solution. Each measurement can only observe damage that occurs within the timeframe of the measurement in a mixture of damaged and undamaged solution. For conventional flow analysis at the critical flux for RNAse A, the model indicates that the dose distribution extends to a maximum of 27 000 Gy, ∼60 times higher than its weighted average dose and 180 times the dose in the centre of the capillary. Thus, the dose distribution is too wide for its average (430 ± 110 Gy) to accurately quantify the fundamental dose limit of the solution itself. A section through the dose distribution for the conventional flow (Fig. 11 ▸) indicates that at the maximum dose before observable damage, almost the entire sample received a much lower dose than the critical average dose as observed by coflow except for the region within 50 µm of the capillary walls. Thus, in conventional measurements the high dose region near the capillary wall prematurely limits the dose deliverable to the whole sample. Quantitative analysis of the dose distribution at the conventional critical flux for RNAse A shows that 90% of the scattering intensity comes from pixels receiving only half or less of their critical dose. In contrast, in coflow measurements the dose distribution is reasonably uniform and is primarily controlled by the cross-sectional beam profile. The maximum dose of 2250 Gy was only 1.5 times the average protein dose (1500 ± 270 Gy) and 1.3 times the dose in the centre of the capillary; hence, coflow should be capable of assessing the critical dose to a similar degree of approximation as static analysis. Unfortunately, Jeffries and coworkers reported critical doses of 250–320 Gy for static RNAse A in PBS, which appear to be more consistent with the conventional measurement and suggest that the coflow value is inaccurate (Jeffries *et al.*, 2015 ▸). However, the doses of Jeffries and coworkers were based on an arbitrarily large definition of the beam-interaction volume that underestimated the absolute dose as observed by SAXS. Recalculating the critical dose of Jeffries and coworkers with the dose rates calculated by Hopkins & Thorne (2016 ▸) and the time to critical dose quoted by Jeffries *et al.* (2015 ▸) gives a value of ∼800 Gy, which is closer to the coflow value. Our analysis using pixel-based scattering intensity weighted average dose modelling and the flux and time to critical dose quoted by Jeffries *et al.* (2015 ▸) indicates a critical dose of 720–920 Gy, similar to the conclusion of Hopkins and Thorne. The difference between coflow and the results of Jeffries and cowokers may be owing to differences in flux calibrations, the finite dose resolutions of our collected data sets, damage kinetic effects that may occur for static measurements but not for coflow, or other experimental factors. While critical dose is an important measure of resistance to radiation damage, we agree with Jeffries and coworkers that of more practical concern is ensuring that routine measurements are accurate (*i.e.* not significantly disaffected by radiation damage) and providing practical means of ensuring this in routine measurement (Jeffries *et al.*, 2015 ▸). The results presented here clearly show that coflow provides a convenient means to achieve this goal under flowing conditions.

Secondly, unlike conventional measurements, coflow showed no evidence of aggregation in the radiation damaged RNase A protein sample. Analysis of the flow velocity distribution for coflow shows that no RNAse A molecule spent more than 0.063 s within the FWHM of the beam at the flow rate used for the measurement, whereas for static measurements the whole observed sample is typically exposed for hundreds of milliseconds to seconds. Each method can only observe radiation damage processes that occur within the timeframe of the measurement. We hypothesise that the lack of aggregation observed for coflow, even at doses sufficient to cause conformation change, indicates that aggregation may be a slow process occurring after the sample has traversed the beam position and is no longer detectable in the SAXS measurement. This is supported by the small dwell time in coflow. The kinetics of mass transport for aggregation in a flowing system may also be different from a static system. Coflow has the capability to provide high doses in short effective measurement times, particularly for high flow rates, high fluxes and small beam sizes, with a narrow dwell-time distribution controlled primarily by the flow rate distribution of the sample stream. Thus, the coflow approach opens new avenues for the study of the kinetics of radiation damage, and the potential to avoid the effects of radiation damage processes that are kinetically slower than the timeframe of the measurement.

Finally, the fact that buffers showed a significant response to the high flux beam in coflow unless protected by an additive such as glycerol is a cautionary tale. Whilst buffer damage requires higher doses than proteins, its effects in conventional flow measurements at lower flux may be subtle and may not always be easily distinguishable from radiation damage to proteins. Therefore, it is prudent to use additives that protect buffers from radiation damage in routine beamline operations.

Coflow provides two additional practical advantages. Firstly, the efficient X-ray dose delivery of coflow is designed to couple with efficient sample loading and injection geometry. This readily allows a large reduction in the minimum required sample volume. Samples as small as 2 µl can be analysed in steady-state flow conditions, and samples between 5 and 10 µl can be analysed routinely, a tenfold improvement over conventional measurements on our previous equipment. Secondly, the physical separation of sample from capillary allows a complete avoidance of capillary fouling by samples, even at doses sufficient to cause severe damage to proteins. These advantages aid routine and robust operations of synchrotron beamlines, and make coflow a convenient and powerful method to study valuable, difficult-to-obtain and unstable samples.

Coflow provides a comprehensive approach to further exploit the full potential of intense synchrotron sources for radiation sensitive solution samples. The rapid establishment of steady-state flow, the ability to use much higher flux per sample volume, the intrinsic robustness against sample fouling and the low sample consumption of coflow are significant improvements. Coflow provides a convenient means of studying radiation damage without of the burden of capillary fouling by the sample, with access to radiation damage kinetics that are inaccessible using conventional methods. The coflow method extends the routine application of SAXS to challenging areas of biology that involve unstable, beam-sensitive and low-volume samples, which are increasingly of interest for health and industrial applications.

## Supplementary Material

Supplementary Figures.. DOI: 10.1107/S2059798316017174/jc5006sup1.pdf
