# Supplementary material for: Improved radiation dose efficiency in solution SAXS using a sheath flow sample environment
Source: Acta Crystallogr D Struct Biol. 2016 Nov 29;72(Pt 12):1254–66. doi: 10.1107/S2059798316017174 (PMC5137223; doi:10.1107/S2059798316017174)
Supplement: Supplementary file 1 [file d-72-01254-sup1.pdf]

STRUCTURAL
BIOLOGY

Volume 72 (2016)

Supporting information for article:

Improved radiation-dose efficiency in solution SAXS using a sheath-flow sample environment

Nigel Kirby, Nathan Cowieson, Adrian M. Hawley, Stephen T. Mudie, Duncan J. McGillivray, Michael Kusel, Vesna Samardzic-Boban and Timothy M. Ryan

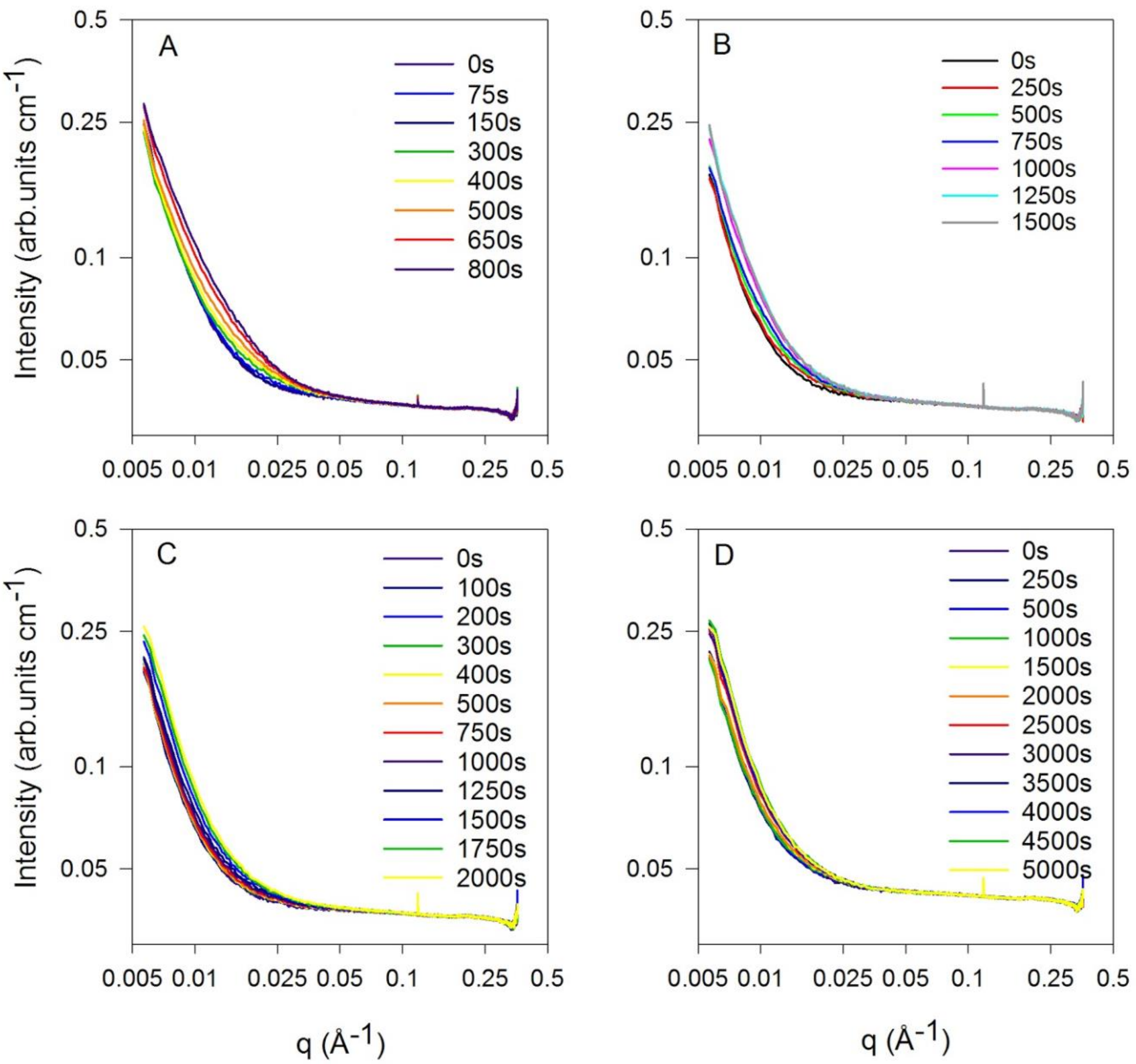

Figure S1 Damage in buffers manifests as increases in intensity at low q. The effect of radiation dose to glycerol-free HEPES (A), Tris.HCL (B), MES (C) and PBS (D) was investigated by continuous X-ray exposure for the time indicated in the respective legend, and acquiring SAXS measurements every 5 seconds.

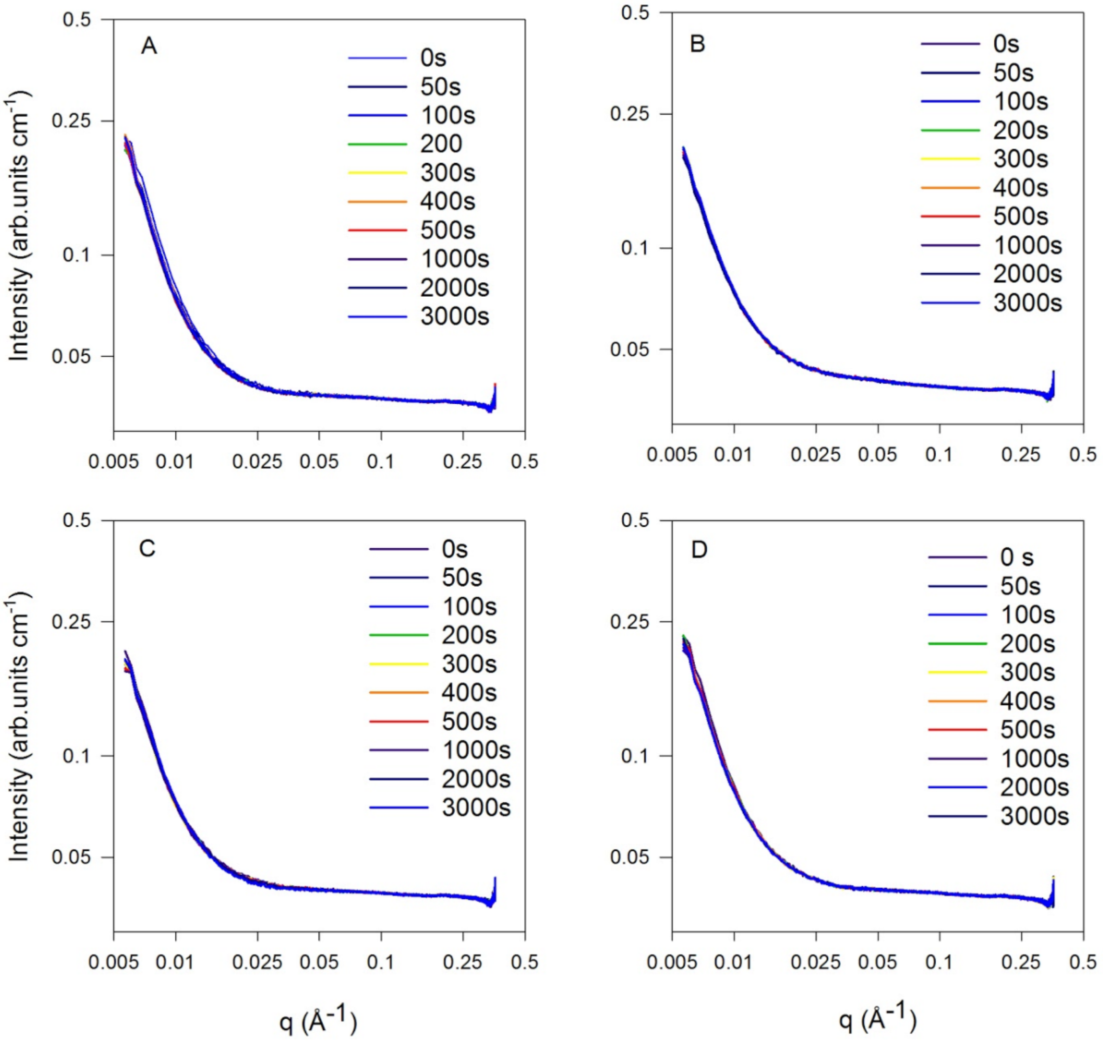

Figure S2 The effect of radiation dose to HEPES (A), Tris.HCL (B), MES (C) and PBS (D) supplemented with 5% glycerol was investigated by applying beam for the time indicated in the respective legend, and acquiring SAXS measurements every 5 seconds.

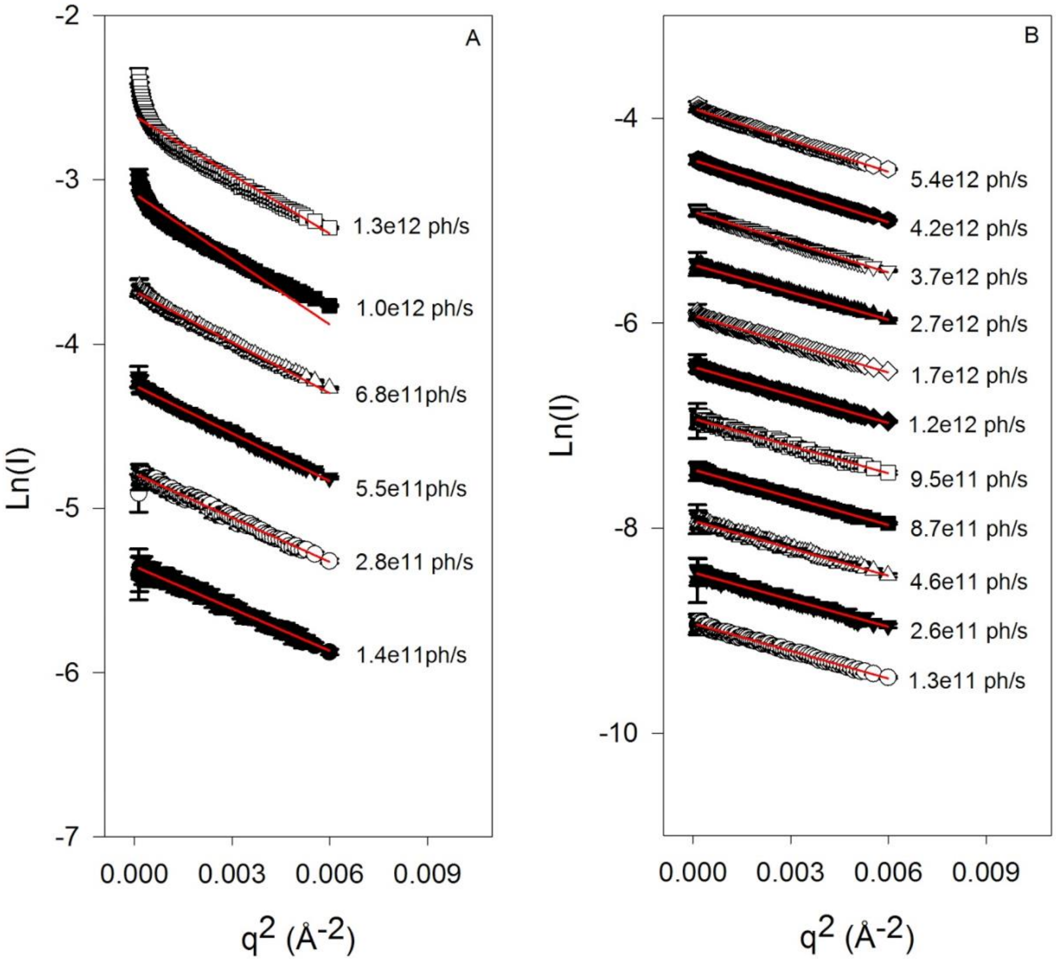

Figure S3 Guinier fits for RNAse A damage. Conventional (A) and coflow analysis (B). The q-range of all Guinier fits was held constant to give an indication that damage has occurred, extending from the lowest q measured to a maximum q equal to 1.3 / R_g, where Rg is the R_g of undamaged RNase A (16.2) (range is 0.011 – 0.08).

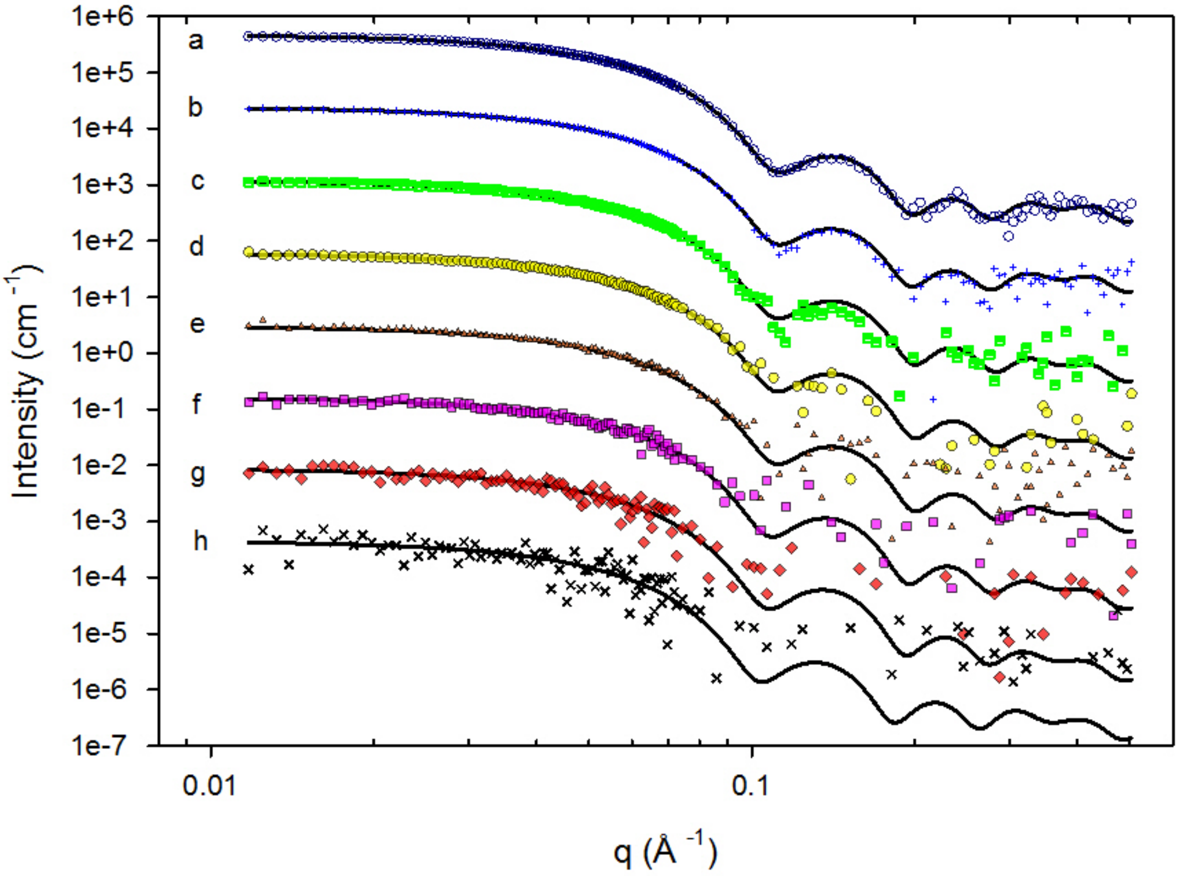

Figure S4 Accuracy of the coflow method. Crysol fits to glucose isomerase concentration series (a) 1.0, (b) 0.50, (c) 0.25, (d) 0.125, (e) 0.0625, (f) 0.0313, (g) 0.0152, (h) 0.0075) mg/mL. The most dilute sample is plotted as measured and successive curves are offset by a factor of 10 for clarity.

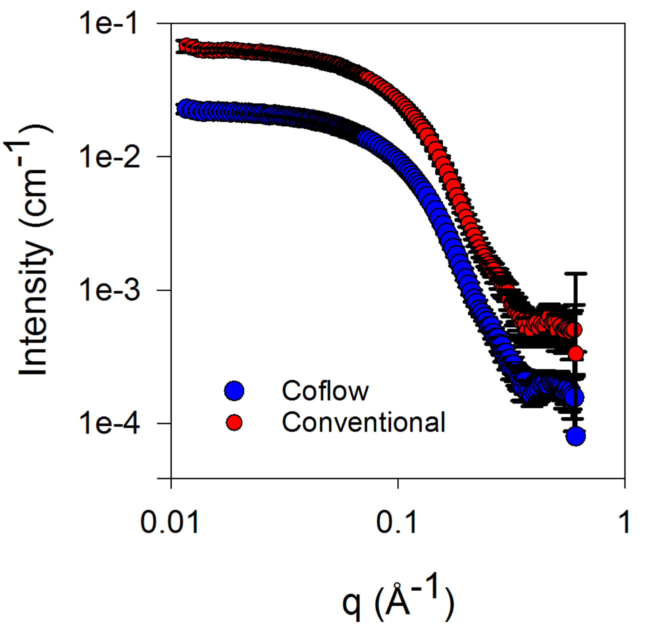

Figure S5 Improvement in data quality. SAXS patterns for 6 mg/mL RNAse A in glycerol-free PBS measured at critical flux at 12 keV. Upper red curve is conventional flowing analysis is for 10 µL of sample flowing at 1 µL/s measured for 9 seconds of sample and 25 seconds of buffer at 2.1 x 10^{11} ph/s. Lower blue curve is coflow for 10 µL with FSFR = 0.33 and total flow rate of 1 µL/s measured at 2.4 x 10^{12} ph/s covering 25 seconds of both sample and buffer. Uncertainties are ± 2 standard errors of mean intensity in each q bin from ScatterBrain.
